# The urinary microbiota of men and women and its changes in women during bacterial vaginosis and antibiotic treatment

**DOI:** 10.1186/s40168-017-0305-3

**Published:** 2017-08-14

**Authors:** Cornelia Gottschick, Zhi-Luo Deng, Marius Vital, Clarissa Masur, Christoph Abels, Dietmar H. Pieper, Irene Wagner-Döbler

**Affiliations:** 1 Research Group Microbial Communication, Helmholtz Centre for Infection Research, Inhoffenstr. 7, 38124 Braunschweig, Germany; 2 Microbial Interactions and Processes Research Group, Helmholtz Centre for Infection Research, Inhoffenstr. 7, 38124 Braunschweig, Germany; 3 Dr. August Wolff GmbH & Co. KG Arzneimittel, Sudbrackstrasse 56, 33611 Bielefeld, Germany

**Keywords:** Healthy urinary microbiome, Urinary microbiota, Urotypes, Bacterial vaginosis, Antibiotic treatment, Vaginal microbiota

## Abstract

**Background:**

The urinary microbiota is similarly complex as the vaginal and penile microbiota, yet its role as a reservoir for pathogens and for recurrent polymicrobial biofilm diseases like bacterial vaginosis (BV) is not clear.

**Results:**

Here, we analysed the urinary microbiota of healthy men and women and compared it with that of women during BV and after antibiotic treatment using next-generation sequencing of the 16S rRNA gene V1-V2 regions. Eight different community types, so called urotypes (UT), were identified in healthy humans, all of which were shared between men and women, except UT 7, dominated in relative abundance by *Lactobacillus crispatus*, which was found in healthy women only. Orally applied metronidazole significantly reduced Shannon diversity and the mean relative abundance of *Gardnerella vaginalis*, *Atopobium vaginae*, and *Sneathia amnii*, while *L. iners* increased to levels twofold higher than those found in healthy women. Although individual urine microbial profiles strongly responded to the antibiotic, the healthy community could not be restored. The correlation between urinary and vaginal fluid microbiota was generally weak and depending on UT and BV status. It was highest in UT 1 in acute BV (59% of samples), but after metronidazole treatment, only 3 out of 35 women showed a significant correlation between their urinary and vaginal microbiota composition.

**Conclusions:**

Urethra and bladder thus harbor microbial communities distinct from the vagina. The high abundance of BV related species in the urine of both men and women suggests that urine may act as a reservoir of pathogens and contribute to recurrence.

**Trial registration:**

ClinicalTrials.gov, NCT02687789

**Electronic supplementary material:**

The online version of this article (doi:10.1186/s40168-017-0305-3) contains supplementary material, which is available to authorized users.

## Background

The dogma of sterile urine and bladder persisted for a long time and was based on the assumption that all bacteria are pathogens and absent in healthy people [1]. This paradigm has shifted, and now the importance of commensal organisms for human health is widely acknowledged. Different body sites have since been studied extensively, but only recently the female urinary microbiota (FUM) attracted attention [2]. Studies have examined the FUM in health and disease focusing on asymptomatic bacteriuria (ABU) [3], interstitial cystitis (IC) [4] and urgency urinary incontinence (UI) [5]. In these studies, first void or midstream urine as well as urine obtained using transurethral catheters was sampled. Another study compared the different urine sampling methods directly and sometimes found the same genera [6]. Thus, it could clearly be demonstrated that in healthy women the bladder is colonized by *Lactobacillus*, *Gardnerella*, *Prevotella*, *Corynebacterium*, *Sneathia* and other genera that are commonly found in bacterial vaginosis [7].

A recent study identified six urotypes in the healthy midstream FUM; they were dominated in abundance by *Lactobacillus*, *Gardnerella*, *Sneathia*, *Staphylococcus* or *Enterobacteriaceae* or consisted of highly diverse microbiota [5]. Inter-individual differences in community composition and diversity were high [4, 8, 9]. It was shown that urinary bacteria are viable, and midstream urine contains a mixture of urinary and genital tract bacteria [6, 10].

The role of the microorganisms colonizing the healthy female bladder for diseases is unclear. No single pathogen could be identified to be causative for asymptomatic bacteriuria, interstitial cystitis or urgency urinary incontinence [3–5]. The same urotypes identified in individuals free of symptoms could also be observed in UI [11]. Nevertheless, differences with regard to *Lactobacillus sp.* which are less abundant in ABU and UI and more abundant in IC could be observed [12].

There are only a few studies on the male urine microbiota (MUM). The healthy MUM is characterized by genera such as *Lactobacillus*, *Sneathia*, *Veillonella*, *Corynebacterium Prevotella*, *Streptococcus* and *Ureaplasma*, and these are also found on urethral swabs [13, 14]. A study of men with and without sexually transmitted infections (STIs) found that bacteria associated with STI but also with vaginal dysbiosis can be found in STI-positive patients [15]. Furthermore, the bacterial composition in urine changes with age in both men and women [16]. So far, no study has sampled male urine directly out of the bladder. No distinct urotypes have been identified in male urine, and no direct comparison between FUM and MUM using high-resolution amplicon sequencing has been conducted so far.

The composition of the urine microbiota in women diagnosed with bacterial vaginosis (BV) has not yet been investigated. BV is the most common vaginal syndrome in women of childbearing age, with a prevalence of up to 30% [17], and it has a 60% recurrence rate in the 12 months after treatment with the standard care antibiotic metronidazole [18]. BV is a disease of the vaginal tract that is characterized by a loss of commensal *Lactobacillus sp.* and a concurrent increase of diversity and pH. It has been hypothesized that the high recurrence rate of BV might be caused by biofilms of BV-associated bacteria like *Gardnerella vaginalis* and *Atopobium vaginae* on vaginal epithelial cells which resist antibiotic treatment and thus continue to reside in the genital tract [19]. As observed with pathogens causing sexually transmitted infections, some commensal bacteria may be transferred from women to men, since bacterial communities in the male urethra and penile skin were strongly correlated with those of the vagina in married couples, but only if the women had BV [20]. A comparison between sexually active and inactive adolescent males showed that BV-associated genera such as *Sneathia*, *Mycoplasma* and *Ureaplasma* were only found in sexually experienced adolescents [14]. Furthermore, biofilms were identified in urine of BV patients and their partners [21] suggesting that sexual transmission of biofilm-associated bacteria may be another reservoir for BV; although, it is not considered a classical sexually transmitted disease.

We previously screened for a compound that could disrupt biofilms of *G. vaginalis* [22] and conducted a randomized clinical trial to test its effectiveness in BV, especially with respect to biofilms in the vaginal fluid and recurrence [23]. We found that most women with BV were biofilm positive and that metronidazole had a high cure rate, leading to a decrease of diversity along with the disappearance of biofilms and symptoms. However, the abundance of *Lactobacillus iners* increased compared to a healthy control group, suggesting that metronidazole might not sustainably cure these women. The diversity of the vaginal microbiota increased before recurrence, but none of the clinical and microbiological parameters, including the presence of biofilms, were significantly correlated with recurrence. Therefore, we considered other sources for recurrence and analysed the urinary microbiota.

Although similar genera and species of bacteria have been identified in the urine of healthy men and women as those present in the vaginal fluid during BV [3, 5, 6, 11, 20, 24–26], a comparison between these three types of microbiota with each other using high-resolution amplicon sequencing has not been conducted yet. It is unclear if the microbiota of the urethra and bladder acts as a reservoir for pathogens and in such a way that contributes to BV recurrence. To address these questions, here, we sequenced the highly conserved 16S rRNA gene V1-V2 regions using urine samples of healthy men and women and of women with acute BV before and after antibiotic treatment with metronidazole. With this approach, it was possible to identify key taxa to the species level and to classify the microbial profiles into urotypes. The effect of BV on the urinary microbiota was investigated and the influence of metronidazole treatment was compared in urine and vaginal fluid.

## Methods

### Study design

Samples for analysis were collected in two studies. Midstream urine samples from 109 women with BV were obtained as part of a clinical trial that tested the efficiency of a newly developed pessary against recurrent BV [23]. In this clinical trial, women were screened for BV by gynecologists based on the Amsel criteria and Nugent Score. Of the 109 screened women with BV, 42 women met the strict criteria for inclusion into the clinical study, of which one was the presence of bacterial biofilms in urine and on vaginal epithelial cells. Women were not included into the study if they had *Herpes simplex*, *Candida sp.* or *Trichomonas sp.* infections; had chronic immunosuppressive diseases or treatment; were pregnant or breastfeeding or were active smokers (more than five cigarettes per day). In addition to midstream urine samples, vaginal fluids of these women were collected and vaginal biofilm was stained and analysed on vaginal epithelial cells. In accordance to the German BV-guideline, acute BV was treated with 2 g of metronidazole (single dose) orally at visit 1. Women were sampled before and after metronidazole treatment. The second sampling point was at least 7 days and up to 28 days after metronidazole treatment. The clinical trial was registered on ClinicalTrials.gov with the identifier NCT02687789 [23]. For study details and results see [23]. In a study performed at the Helmholtz Centre for Infection Research in Braunschweig, Germany, midstream urine samples were collected from healthy employees as part of their routine examination at work, namely 31 healthy men and 49 healthy women. Only women that reported to be free of BV were included into this study. The study protocols were approved by the local ethics committees (Niedersächsische Landesärtzekammer, Hannover and Bayerische Landesärtzekammer, München) and written consent was obtained from all participants.

### Sample collection, transport and DNA extraction

Midstream urine samples used for sequencing were immediately transferred to a urine collection and preservation tube (Norgen Biotek Corp., Canada) holding 50 ml and stored at room temperature until further processing. DNA was extracted from 15 ml urine using the peqGOLD Tissue DNA Kit (Peqlab, Germany). Urine was centrifuged at 7000 rpm for 15 min, and DNA was extracted with pretreatment and modification as described [23]. Briefly, urine was centrifuged at 7000 rpm for 15 min (vaginal fluid 13,000 rpm for 1 min). Pellets were resuspended in 700 μl lysis buffer, 15 μl RNase and 20 μl proteinase K. This suspension was then added to 0.5 g of silica beads which were covered with 500 μl cooled phenol. For cell lysis, the bead-suspension mix was shaken at 5 m/s for 1 min in three intervals which were 2 min apart using the MO-BIO PowerLyzer™ (Mo Bio Laboratories, USA). After centrifugation for 1 min at 13000 rpm, the upper phase containing the DNA was further processed according to the manufacturer’s instructions starting with DNA binding. The mean DNA yield was 5.9 ng/μl and varied from 2 ng/μl to 14.3 ng/μl.

### Preparation of 16S rRNA gene amplicon libraries, sequencing and data processing

Amplicon library preparation for high throughput sequencing of urine samples on a MiSeq Illumina platform (280 bp paired-end chemistry) was performed as previously described [27]. DNA extraction controls as well as positive and negative controls for PCR reactions were included. Because extraction controls showed no PCR products, they were not included for sequencing. Barcoded amplicons of the hypervariable 16S rRNA gene V1-V2 regions were sequenced, and a total of 10,219,820 raw reads obtained. 4,689,672 reads were obtained after quality filtering, primer trimming and merging pairs. Clustering was performed as previously described [23] and resulted in 497 operational taxonomic units (OTUs) and a total of 4,389,556 reads. Taxonomic assignment was performed using standalone blast against the vaginal 16S rDNA reference database (STIRRUPS) [28]. Preparation of 16S rRNA gene amplicon libraries and sequencing of vaginal fluid samples was performed independent of urine samples, but both were processed simultaneously in the same bioinformatics pipeline.

### Statistical analysis of the microbiota

OTU data were rarefied to 2196 reads, and all subsequent analyses were performed on this dataset. Resampling efficiency was determined based on the standard error (standard deviation/mean) after resampling 20 times. Rarefaction curves were obtained with the vegan package in the R environment. Boxplots, dominance plots, shade plots and bar plots were created in PRIMER7 with the PERMANOVA+ add-on software (159 PRIMER-E, 1). The mean relative abundance was calculated by adding all reads for each OTU and dividing them by the number of samples. Shannon index (H′) was determined on the OTU level in PRIMER7 and mean values and standard deviations were calculated. For statistical tests, the Wilcoxon rank sum test was used for unpaired data and the paired Wilcoxon rank-sum test for paired data in R. For the principle coordinate analysis (PCO), a resemblance matrix was generated using the Bray-Curtis coefficient in PRIMER-E7. Clustering of samples was performed based on the Euclidean distance using the Ward clustering method in R. Spearman correlations between urine and vaginal fluid samples, corresponding *p* value calculations and Bonferroni correction were also performed in R.

## Results

### Subjects characteristics

The median age of the healthy men was 29 years and ranged between 23 and 58 years. The median age of the healthy women was also 29 years and ranged between 19 and 62 years. The mean age of women with BV was 31 years old and their age ranged between 18 and 51 years (Table 1). None of the healthy participants indicated to have BV or periodontitis, and 12% had taken antibiotics in the 10 days before sample contribution which did not notably affect the microbial communities of these study participants (Additional file 1: Figure S1). In the BV study group, 23.3% had previously suffered from BV and 2.3% had periodontitis. Eighty-four percent of patients were of Caucasian origin and 12% were of African descent. None of the BV patients had taken antibiotics in the 10 days before the BV incident.

**Table 1:** Overview of the different study groups

Study group	Number of participants	Median age (range)
Healthy men	31	29 (23–58) years
Healthy women	49	29 (19–62) years
Women with BV/receiving treatment	109/43	31 (18–51) years
Women after treatment	43	31 (18–51) years

### Taxonomic affiliation of reads

After abundance filtering, 497 OTUs and a total of 4,389,556 V1-V2 ribosomal DNA reads (mean per sample = 16,502 ± standard deviation = 10,396 reads) were obtained. Thirty-eight percent of the sequences could be assigned to the species level, 42% to the genus level and 20% to the family level or higher taxa. A full OTU table is available in Additional file 2: Table S1. A rarefaction analysis was performed and showed that most of the samples had or had almost reached saturation (Additional file 3: Figure S2). Six samples of the BV study group were excluded due to too little sequencing reads (mean per sample = 694 ± SD = 605 reads). Because the sequencing depth was then between 2196 and 69,765 reads, the dataset was resampled to determine the error introduced by resampling. Four samples with different sequencing depths (5042 reads; 17,477 reads; 43,494 reads and 69,765 reads) were resampled 20 times (Additional file 4: Figure S3). The standard error was very low in high abundant OTUs and up to 5% in low abundant OTUs. Because this study does not focus on rare OTUs, resampling this dataset once was sufficient.

### The male urinary microbiota clustered as part of the female urinary microbiota which was different in health and BV

**Fig. 1:**
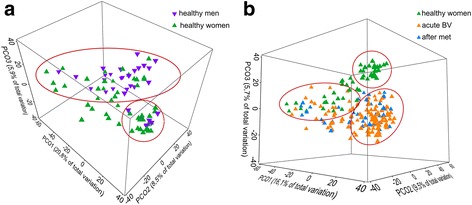
Principle coordinate analyses of the urinary microbiota of healthy men and women (**a**) and women during acute BV, after metronidazole treatment, and in health (**b**)

The healthy male urinary microbiota (MUM) clustered in two distinct parts of the female healthy urinary microbiota (FUM) (Fig. 1a, red circles). Thus, it was not possible to differentiate between MUM and FUM based on the microbiota composition. The FUM was additionally characterized by bacterial communities that could not be found in the MUM. Comparing the urine microbiota of healthy women with that of women with acute BV showed a separation into two different clusters and an overlapping region where a subgroup of samples from healthy as well as symptomatic women were located (Fig. 1b, red circles). Interestingly, samples taken after metronidazole treatment mainly remained in the BV cluster and no shift towards the “health” cluster could be observed (Fig. 1b). Oral metronidazole treatment apparently did not shift the urine bacterial community. While the vaginal microbiota was massively changed by the antibiotic [23], the FUM could not be shifted to the healthy cluster by metronidazole treatment (Additional file 5: Figure S4).

### Metronidazole treatment of BV results in loss of diversity of the female urine microbiota

**Fig. 2:**
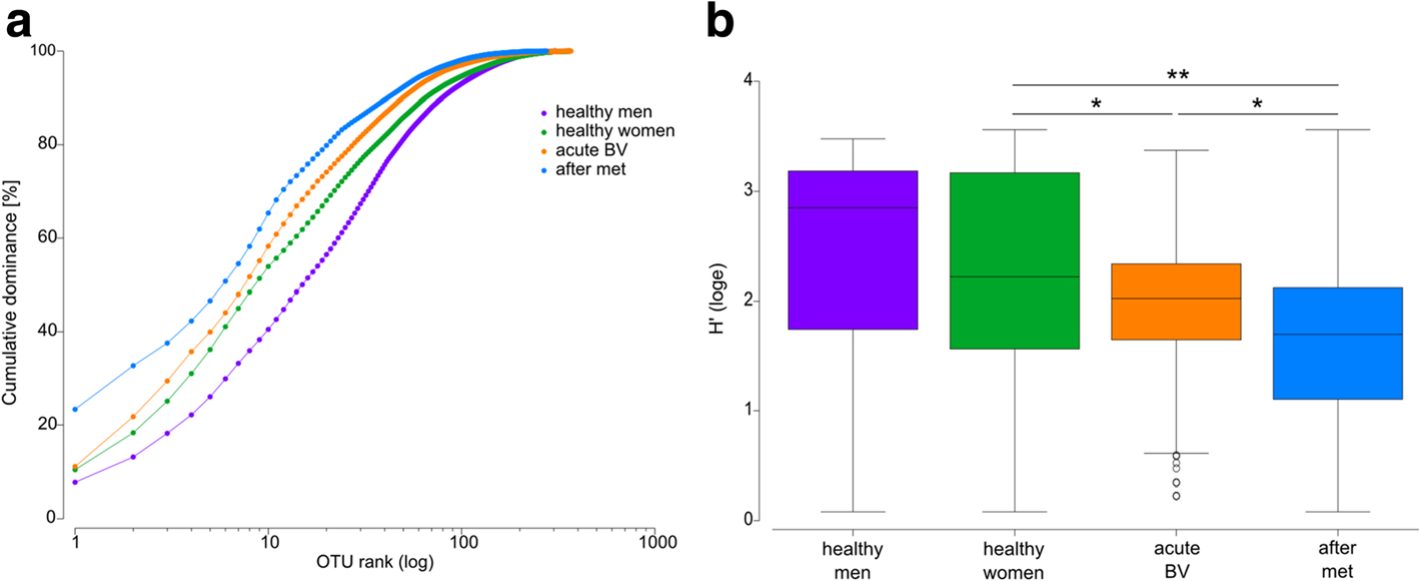
Rank abundance and Shannon diversity of urinary microbiota. **a** Dominance plot of cumulated samples from healthy men and women and women during acute BV and after metronidazole treatment. **b** Shannon indices of all groups. Mean and quartile range are shown. *Asterisks* indicate significant (*p* < 0.01) differences

The urine microbiota diversity was clearly different in the various treatment groups analysed here: Diversity was largest in healthy men, followed by healthy women, then by women with acute BV and was lowest in women after metronidazole treatment of BV (Fig. 2). Interestingly, the urine microbiota of healthy men, although nested in the healthy female microbiota cluster, had a higher Shannon diversity but lower alpha-diversity than that of women, indicating that less OTUs were present, but their abundance was more evenly distributed. In samples of healthy men, 308 OTUs were found and 16 OTUs were needed to cover 50% of cumulative abundance, while 365 OTUs were identified and 9 OTUs needed to cover 50% of cumulative abundance in samples of healthy women. The difference in alpha-diversity and rank abundance in the urine of woman with or without BV was minimal, the latter contained 368 different OTUs and 8 OTUS were needed for 50% abundance. Diversity collapsed after metronidazole treatment, since only 275 OTUs were identified and 6 OTUs were needed to cover half of the total abundance. This was similarly shown by the Shannon index (H′) which was highest in healthy men (H′ = 2.45 ± 0.94) and women (H′ = 2.27 ± 0.91; Fig. 2b). Diversity in the urine microbiota decreased in women with acute BV (H′ = 1.89 ± 0.64), in contrast to the situation in vaginal fluid, where BV is associated with a massively increased diversity and vaginal health is associated with low diversity. This shift in diversity was significant (*p* = 0.014). Treatment with metronidazole resulted in a further significant decrease of diversity in urine (H′ = 1.66 ± 0.69, *p* = 0.042). Microbial urinary communities of women were therefore also significantly less diverse after metronidazole treatment than in healthy women (*p* = 0.0008). These findings can be interpreted as the result of an increased relative abundance of BV-associated OTUs during BV which thereby diminished diversity. After metronidazole treatment, growth of the metronidazole-resistant OTUs might have further reduced microbial diversity when BV-associated OTUs had already been eradicated (Additional file 6: Figure S5).

### Eight different urotypes were identified in the urinary microbiota

**Fig. 3:**
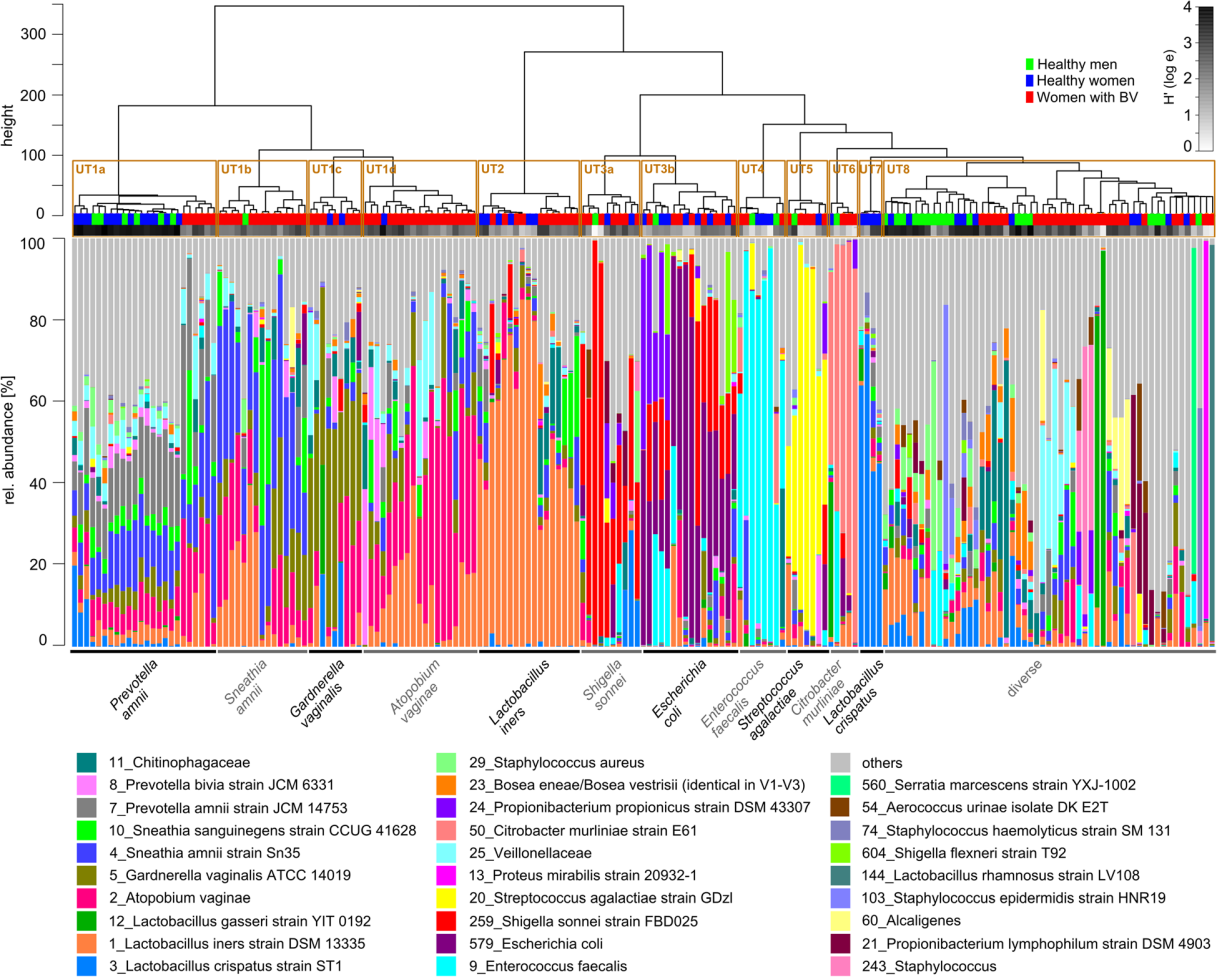
Individual urinary microbiota of healthy men and women and women during acute BV clustered according to urotypes. Clustering of samples was performed based on the Euclidean distance using the Ward clustering method in R. The 19 most abundant OTUs are shown and all others (<1.2% rel. abundance each) are summarized as “others”. Urotypes (UTs) 1–8 with subtypes are indicated in the *boxes* above the relative abundance plot. *Colored boxes* above the abundance plot indicate sample source and *grey boxes* decode diversity (H′)

The urinary microbiota of healthy men and women and of women with BV clustered into eight different urotypes (Table 2) (Fig. 3). Urotype (UT) 1 was comprised of four sub-clusters (a through d) and UT 3 of two sub-clusters (a and b). All clusters contained samples from men and women, in acute BV and after metronidazole treatment, with the exception of UT 2, which was only found in women (both during BV and after metronidazole treatment), and UT 7 which was only found in healthy women.

**Table 2:** Overview of all urotypes and their characteristics

Urotype before treatment	Number of samples	Composition of samples			Characteristic OTUs	Diversity (mean H′ ± SD)
la	24	M 25%	F 25%	BV 50%	*Prevotella amnii*	2.44 ± 0.61
lb	15	M 7%	F 0%	BV 93%	*Sneathia amnii*	
lc	9	M 0%	F 22%	BV 78%	*Gardnerella vaginalis*	
Id	19	M 0%	F 5%	BV 95%	*Atopobium vaginae*	
2	17	M 0%	F 35%	BV 65%	*Lactobacillus iners*	1.79 ± 0.63
3a	10	M 10%	F 20%	BV 70%	*Shigella sonnei*	1.63 ± 0.63
3b	16	M 6%	F 44%	BV 50%	*Escherichia coli*	
4	8	M 25%	F 38%	BV 38%	*Enterococcus faecalis*	1.18 ± 0.67
5	5	M 14%	F 14%	BV 72%	*Streptococcus agalacticie*	1.45 ± 0.88
6	6	M 20%	F 20%	BV 60%	*Citrobacter murliniae*	0.85 ± 0.43
7	4	M 0%	F 100% BV 0%		*Lactobacillus crispatus*	2.05 ± 0.74
8	55	M 33%	F 18%	BV 49%	diverse	2.29 ± 0.81

*M* healthy men, *F* healthy women, *BV* women with BV, *H′* Shannon index, *SD* standard deviation

UT 1 was characterized by large diversity (H′_UT1_ = 2.44 ± 0.61) and high relative abundance of the BV-associated OTUs *Prevotella amnii*, *Sneathia amnii*, *Gardnerella vaginalis* and *Atopobium vaginae*, which defined four sub-clusters. UT 1a was characterized by high relative abundance of *P. amnii* and lower relative abundances of *S. amnii*, *G. vaginalis*, *A. vaginae*, *Sneathia sanguinegens*, *L. iners* and *Lactobacillus crispatus*. It showed two distinct sub-clusters, one that contained only samples of healthy men and women and one containing only samples of women with BV. These two sub-clusters showed a marked difference in diversity, e.g. a relatively high diversity in healthy men and women compared to a lower diversity in women with BV (H′_UT1a-HEALTH_ = 3.27 ± 0.12 vs. H′_UT1a- BV_ = 2.13 ± 0.46, *p* = 0.0015). UT 1b was characterized by a high relative abundance of *S. amnii*, and was further characterized by abundant *L. iners*, *A. vaginae*, *G. vaginalis* and *S. sanguinegens* and low abundant *L. crispatus* and *P. amnii*. UT 1c was defined by a high relative abundance of *G. vaginalis* and further characterized by abundant *A. vaginae* and *L. crispatus* and lower relative abundance of *Prevotella sp.* and *Sneathia sp*. UT 1d was defined by a high relative abundance of *A. vaginae* in relation to the other clusters within UT 1 and was, like UT 1b, characterized by a higher relative abundance of *L. iners*. UT 1b, c and d were dominated in relative abundance by samples from women with BV, but a few samples from healthy men and women were also present. UT 2 was mainly composed of *L. iners* and had a moderate diversity (H′_UT2_ = 1.79 ± 0.63). It was the dominant OTU in this UT and in some samples accompanied by BV-associated bacteria like *S. amnii* and *S. sanguinegens*. UT 2 was only found in women, both with and without BV. UT 3 was characterized by *Enterobacteriaceae* and a moderate diversity (H′_UT3_ = 1.63 ± 0.63). This urotype could be sub-clustered into urotypes 3a and 3b according to the relative abundances of *Sh. sonnei* (UT 3a) and *Escherichia coli* (UT 3b), respectively, and occurred in both health and disease, in both men and women. UTs 3a and 3b were further characterized by abundant *Propionibacterium propionicus* or *Enterococcus faecalis*. UT 4 was strongly dominated in relative abundance by *Enterococcus faecalis* and therefore had a low diversity (H′_UT4_ = 1.18 ± 0.67), UT 5 was mainly composed of *Streptococcus agalactiae* and had a moderate diversity (H′_UT5_ = 1.45 ± 0.88) and UT 6 was highly dominated by *Citrobacter murliniae* and therefore also had a very low diversity (H′_UT6_ = 0.85 ± 0.43). All of those urotypes were mixed, i.e. they contained samples of healthy men and women and of women with BV. UT 7 was dominated in relative abundance by *L. crispatus*, had a high diversity (H′_UT7_ = 2.05 ± 0.74) and was only found in samples of healthy women. UT 8 was a highly diverse (H′_UT8_ = 2.29 ± 0.81) mixed cluster containing samples from healthy men and women as well as of women with BV and characterized by a number of sub-clusters which contained *L. crispatus* and *L. iners*, *Lactobacillus gasseri*, *Chitinophagaceae* or *Veillonellaceae.*

Therefore, no consistent difference in urotypes was observed between healthy men and women and women with BV except for UT 7, which was comprised of samples from healthy women only as was previously observed. Samples of healthy individuals, both men and women, clustered mainly in UT 1a (75%), UT 4 (62%) and UT7 (100%).

### Individual microbial urinary profiles responded strongly to metronidazole treatment

Strong shifts of microbiota composition were observed in almost every woman after metronidazole treatment (Fig. 3a) (Additional file 4: Figure S4), and the cumulative data show that all of the abundant taxa were affected (Fig. 3b). However, in spite of these shifts in individual microbial profiles, women with acute BV could not be separated from those after metronidazole treatment in a PCO (Fig. 1b). Clustering of microbiota composition revealed that certain urotypes persisted after metronidazole treatment, such as UTs 1, 2 and 3, and an additional urotype was identified (Fig. 3c). This UT 9 was defined by high relative abundance of an OTU of *Chitinophagaceae* and further characterized by the presence of *L. iners* and *L crispatus*. It was nested within UT 8 (before metronidazole treatment). Sequencing depth and DNA concentration after extraction of those samples with high *Chitinophagaceae* abundance were analysed due to the risk of it being a contaminant [29]. Read numbers were below average but not at the bottom (median of 8757 reads compared to 17,187 reads after abundance filter) and above average for the DNA yield (median of 9.2 ng/μl compared to 5.9 ng/μl).

We compared those urotypes that were present before and after metronidazole treatment for enrichment of certain OTUs using LEfSe (Fig. 3d). In UT 1, no significant difference was found regarding *P. amnii*, *S. amnii*, *G. vaginalis* and *A. vaginae*, those OTUs that are characteristic of UT 1. However, metronidazole treatment resulted in an increase of other BV-associated OTUs, such as BVAB2, BVAB3 and Megasphaera. In UT 2, the urotype that is mainly composed of *L. iners*, this species increased after antibiotic treatment, while *G. vaginalis* decreased. In UT 3, characterized by *Enterobacteriaceae*, an increase of a different *Enterobacteriaceae* OTU, classified as *Shigella dysenteriae*, was observed. Here, too, the relative abundance of *G. vaginalis* was reduced by antibiotic treatment. Thus, metronidazole treatment resulted in a decrease of *G. vaginalis* in UT 2 and UT 3, but not UT 1, and a concomitant increase of other bacteria, particularly *L. iners*, BVAB2, BVAB3, Megasphaera, and a different Enterobacteriaceae OTU assigned to *Shigella dysenteriae*.

### Relationship between the microbiota of vaginal fluid and urine in BV

**Fig. 4:**
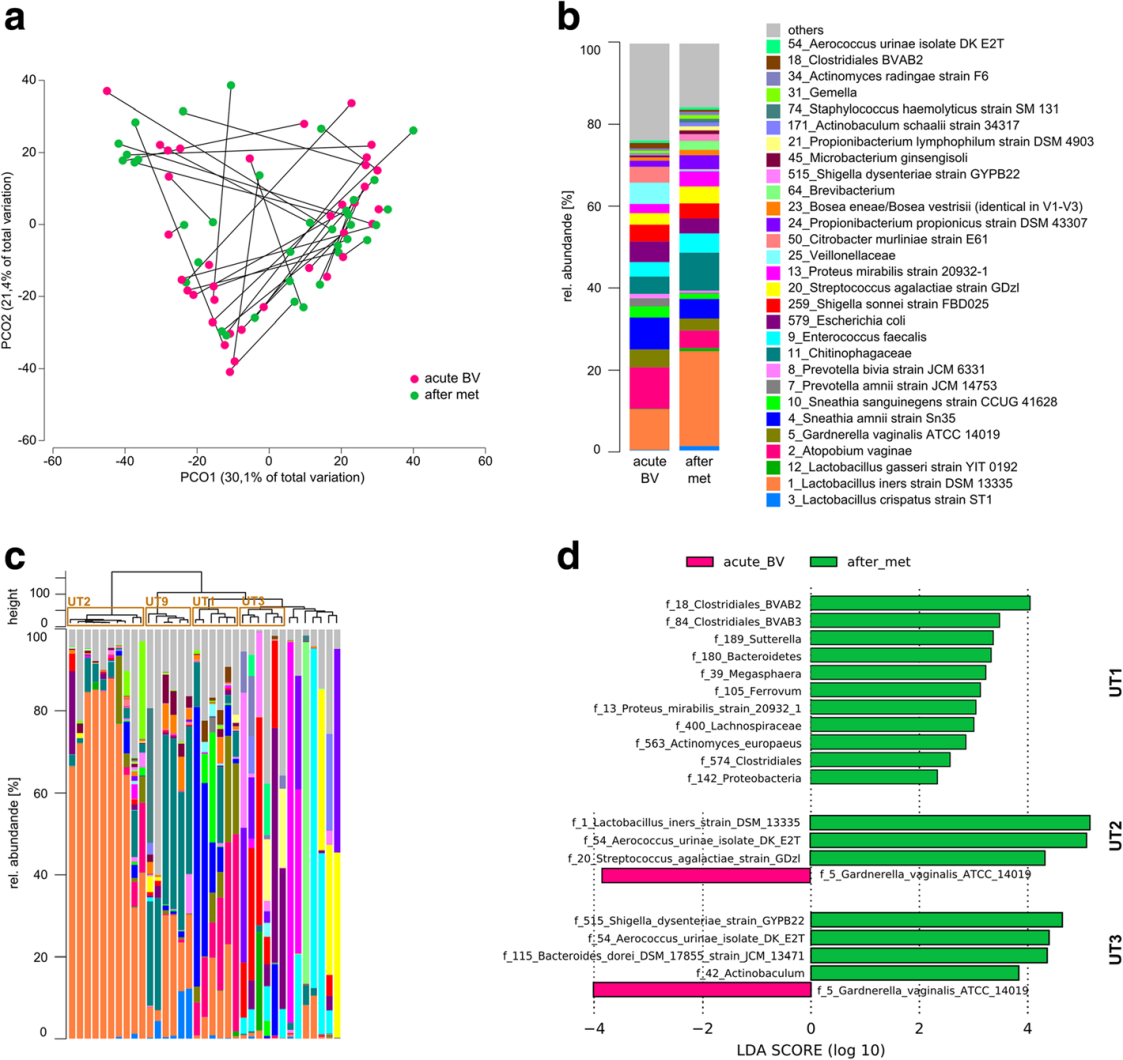
The urinary microbiota during acute BV and after metronidazole treatment. **a** Principle coordinates analysis of urine samples of women with BV before (*red*) and after (*green*) metronidazole treatment. Samples from the same women are connected by a *black line*. **b** Mean relative abundance of urine microbiota during acute BV and after metronidazole treatment. The 19 most abundant OTUs are shown and the remaining OTUs are summarized as “others”. **c** Clustering of urine microbiata after metronidazole treatment. Clustering of samples was performed based on the Euclidean distance using the Ward clustering method in R. Urotypes are indicated. **d** LefSe analysis of urine microbiota belonging to UT1, UT2, and UT3 before and after metronidazole treatment

Since the midstream urine samples analysed here can be expected to contain vaginal fluid microbiota to a certain extent, we compared urine samples before and after metronidazole treatment with vaginal fluid samples of the same patients and at the same time points. Figure 4a shows that a subset of about 40% of the samples clustered together despite the differences in BV status (acute BV or after metronidazole treatment) and sample source (vaginal fluid or urine). The remaining samples were grouped according to source (urine or vaginal fluid), but not according to BV status (acute BV or after metronidazole treatment).

To understand these clusters, we analysed the correlation of urine microbial profile with vaginal fluid microbial profile in the same patient and separated the samples according to UT and considered only those that were significantly correlated (*p* after Bonferroni correction ≤0.01, Table 3) (Additional file 5: Figure S5). All significant correlations had a correlation coefficient *r* ≥ 0.6. In UT 1, the majority of the urine and vaginal fluid samples were significantly correlated with each other (59%). In all other urotypes, none or just a few vaginal fluid samples correlated with the respective urine sample. After treatment, two of the six patients in UT 1 and one in UT 2 showed a correlation between vaginal fluid and urine microbiota, but none of the urine samples from other urotypes correlated with vaginal fluid microbiota composition. These data show that vaginal fluid and urine are most similar when the communities are dominated in relative abundance by bacteria like *P. amnii*, *S. amni*, *G. vaginalis* or *A. vaginae*. However, most urine microbiota were not correlated with their vaginal fluid counterpart, suggesting that different microbiota are located in bladder and urethra compared to the vagina.

**Table 3:** Correlation of vaginal and urine microbiota of the same women

UT	Sample ID	*r*	*p* value	FDR	Sign, corr >0.6	% Corr. samples
Acute BV						
1	05-017a	0.87	1.9E-13	2.5E-11	*	58.97
	13-018a	0.84	6.1E-12	7.7E-10	*	
	13-012a	0.84	7.0E-12	8.9E-10	*	
	05-018a	0.83	2.0E-11	2.5E-09	*	
	06-001a	0.82	6.2E-11	7.9E-09	*	
	17-00la	0.81	1.7E-10	2.2E-08	*	
	13-005a	0.80	2.8E-10	3.5E-08	*	
	13-019a	0.80	50E-10	6.4E-08	*	
	06-002a	0.78	2.4E-09	3.1E-07	*	
	06-009a	0.76	7.0E-09	8.9E-07	*	
	08-003a	0.76	l.0E-08	1.3E-06	*	
	08-002a	0.75	1.5E-08	1.9E-06	*	
	13-014a	0.74	2.7E-08	3.4E-06	*	
	13-007a	0.74	3.3E-08	4.1E-06	*	
	05-016a	0.73	5.3E-08	6.8E-06	*	
	05-004a	0.73	8.1E-08	1.0E-05	*	
	06-010a	0.72	l.2E-07	1.6E-05	*	
	08-008a	0.70	4.3E-07	5.4E-05	*	
	05-005a	0.68	1.2E-06	1.5E-04	*	
	13-017a	0.68	l.3E-06	1.6E-04	*	
	08-005a	0.63	1.1E-05	1.4E-03	*	
	05-014a	0.62	1.3E-05	1.6E-03	*	
	03-003a	0.61	2.7E-05	3.5E-03	*	
	05-008a	0.57	8.7E-05	1.1E-02		
	13-010a	0.54	3.0E-04	3.8E-02		
	08-001a	0.53	3.5E-04	4.5E-02		
	13-003a	0.50	8.4E-04	1.1E-01		
	04-004a	0.48	l.5E-03	1.9E-01		
	08-006a	0.47	1.7E-03	2.2E-01		
	15-006a	0.39	l.2E-02	1.0E + 00		
	13-016a	0.35	2.4E-02	1.0E + 00		
	04-006a	0.34	2.8E-02	l.0E + 00		
	13-015a	0.33	3.3E-02	1.0E + 00		
	05-015a	0.31	5.2E-02	1.0E + 00		
	06-004a	0.31	5.2E-02	l.0E + 00		
	03-004a	0.30	6.0E-02	1.0E + 00		
	05-002a	0.23	1.5E-01	1.0E + 00		
	05-019a	0.07	6.7E-01	l.0E + 00		
	15-002a	-0.17	3.0E-01	1.0E + 00		
2	13-006a	0.81	1.0E-10	1.3E-08	*	20.00
	15-006b	0.63	8.2E-06	1.0E-03	*	
	06-005a	0.55	2.2E-04	2.8E-02		
	06-007a	0.54	3.1E-04	3.9E-02		
	05-007a	0.44	4.1E-03	5.2E-01		
	13-024a	0.42	6.6E-03	8.4E-01		
	15–001a	0.32	4.1E-02	1.0E + 00		
	13-008a	0.27	8.8E-02	l.0E + 00		
	05–02 la	0.18	2.5E-01	1.0E + 00		
	03-005a	0.15	3.5E-01	1.0E + 00		
3	13-011a	0.71	2.0E-07	2.5E-05	*	23.08
	05-020a	0.68	8.4E-07	1.1E-04	*	
	01-004a	0.66	2.9E-06	3.6E-04	*	
	13-025a	0.48	1.4E-03	1.8E-01		
	08-007a	0.46	2.4E-03	3.0E-01		
	07-001a	0.32	4.3E-02	1.0E + 00		
	15-003a	0.30	5.3E-02	1.0E + 00		
	05-013a	0.26	1.0E-01	l.0E + 00		
	05-006a	0.23	1.6E-01	1.0E + 00		
	13-020a	0.16	3.2E-01	1.0E + 00		
	05-009a	0.10	5.3E-01	l.0E + 00		
	15-005a	0.00	9.8E-01	1.0E + 00		
	15-004a	-0.11	4.8E-01	1.0E + 00		
4	13-035a	0.75	2.0E-08	2.6E-06	*	33.33
	17-002a	0.55	2.2E-04	2.7E-02		
	12-002a	-0.04	7.8E-01	1.0E + 00		
5	01-002a	0.56	l.6E-04	2.0E-02		0
	04-005a	0.46	2.4E-03	3.1E-01		
	12-003a	0.40	9.5E-03	l.OE + OO		
6	17-003a	0.34	2.9E-02	l.0E + 00		0
	13-026a	0.11	4.9E-01	l.OE + OO		
8	13-023a	0.86	3.5E-13	4.4E-11	*	27.27
	06-008a	0.78	2.lE-09	2.7E-07	*	
	15-009a	0.78	2.6E-09	3.3E-07	*	
	13-001a	0.76	9.2E-09	1.2E-06	*	
	17-004a	0.74	2.4E-08	3.1E-06	*	
	03-002a	0.74	2.7E-08	3.4E-06	*	
	13-004a	0.56	l.4E-04	1.7E-02		
	04-007a	0.52	5.3E-04	6.7E-02		
	04-00la	0.50	9.8E-04	1.3E-01		
	13-022a	0.47	2.0E-03	2.5E-01		
	05-001a	0.45	3.1E-03	4.0E-01		
	13-009a	0.43	5.4E-03	6.9E-01		
	13-013a	0.32	3.8E-02	l.OE + OO		
	04-008a	0.31	5.0E-02	l.OE + OO		
	05-010a	0.31	5.0E-02	l.OE + OO		
	03-006a	0.27	8.8E-02	l.OE + OO		
	15-007a	0.20	2.1E-01	l.OE + OO		
	13-021a	0.15	3.5E-01	l.OE + OO		
	05-011a	0.07	6.7E-01	l.OE + OO		
	05-012a	0.01	9.6E-01	l.OE + OO		
	04-003a	-0.03	8.5E-01	l.OE + OO		
	05-003a	−0.10	5.5E-01	l.OE + OO		
After metronidazole						
1	06–00lb	0.76	5.9E-09	7.5E-07	*	33.33
	07-001b	0.72	8.6E-08	1.1E-05	*	
	05-013b	0.50	9.9E-04	1.3E-01		
	13-004b	0.23	1.5E-01	l.OE + OO		
	05-001b	0.20	2.0E-01	l.OE + OO		
	05-011b	0.19	2.4E-01	l.OE + OO		
2	12-003b	0.65	5.0E-06	6.3E-04	*	10.00
	01-004b	0.49	1.2E-03	1.5E-01		
	05-004b	0.46	2.5E-03	3.2E-01		
	06-010b	0.44	3.8E-03	4.9E-01		
	13-019b	0.31	4.5E-02	l.OE + OO		
	04-001b	0.29	6.5E-02	l.OE + OO		
	13-023b	0.25	1.2E-01	l.OE + OO		
	05-006b	0.17	3.0E-01	l.OE + OO		
	06-004b	-0.03	8.4E-01	l.OE + OO		
	04-006b	-0.12	4.7E-01	l.OE + OO		
3	13-022b	0.49	1.1E-03	1.4E-01		0
	13-025b	0.37	1.9E-02	l.OE + OO		
	05-008b	0.36	2.1E-02	l.OE + OO		
	05-012b	0.35	2.7E-02	l.OE + OO		
	17-004b	0.31	4.5E-02	l.OE + OO		
	05-017b	0.22	1.6E-01	l.OE + OO		
9	05-021b	0.45	2.9E-03	3.7E-01		0
	05-014b	0.31	5.1E-02	l.OE + OO		
	05-020b	0.25	1.2E-01	l.OE + OO		
	08-005b	0.22	1.6E-01	l.OE + OO		
	08-006b	0.12	4.5E-01	l.OE + OO		
	08-003b	0.11	5.0E-01	l.OE + OO		
−	06-005b	0.51	6.5E-04	8.3E-02		0
	13-015b	0.29	6.7E-02	l.OE + OO		
	13-026b	0.26	9.5E-02	l.OE + OO		
	12-002b	0.26	9.7E-02	l.OE + OO		
	13-001b	0.20	2.2E-01	l.OE + OO		
	06-002b	0.08	6.2E-01	l.OE + OO		
	13-035b	−0.16	3.1E-01	l.OE + OO		

*UT* urotype, *r* Spearman correlation coefficient, *FDR* Bonferroni-corrected *p* value

* significant at p<0.01 after Bonferroni correction

We then analysed the mean relative abundance of OTUs in urine and vaginal fluid samples in BV before and after treatment with metronidazole and compared it to the healthy FUM. Figure 4b shows that the BV-associated bacteria characteristic for UT 1 (*P. amnii*, *S. amni*, *G. vaginalis* and *A. vaginae*) were abundant in both urine and vaginal fluid in BV. This finding is in accordance with the high correlation between urine and vaginal fluid microbiota in BV before antibiotic treatment for those patients whose urinary microbiota belongs to UT 1 (Table 3). Additionally, two OTUs belonging to *Veillonellaceae* were also abundant both in urine and in vaginal fluid in BV. All of those OTUs were strongly reduced by metronidazole treatment in both types of samples. OTUs that were abundant in vaginal fluid samples, but not in urine, were *S. sanguinegens* and several OTUs belonging to the genus *Prevotella*.

**Fig. 5:**
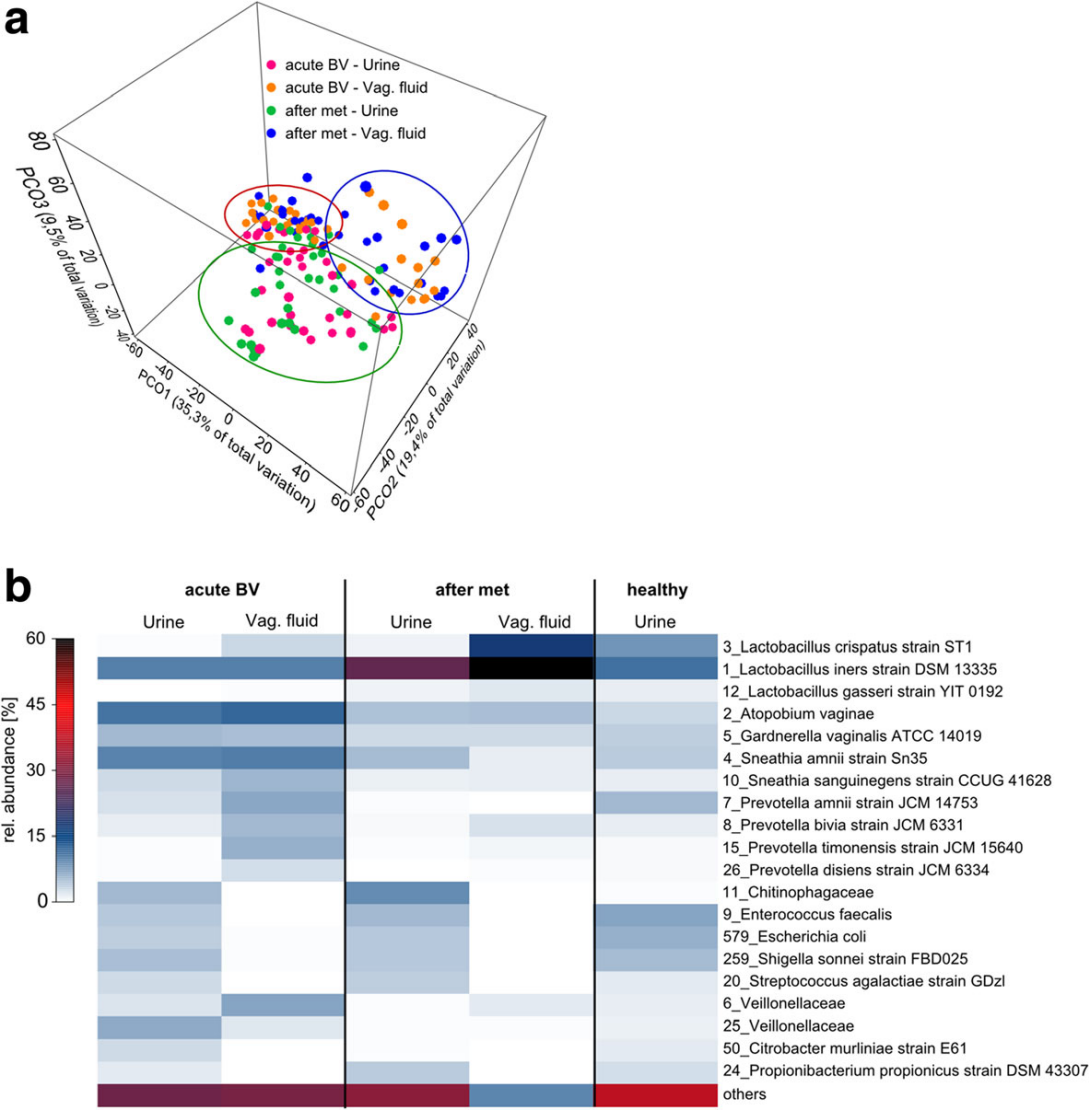
Comparison between urine and vaginal fluid microbiota in acute BV and after metronidazole treatment. **a** Principal coordinates analysis. **b** Heatmap of the mean relative abundance of the 20 most abundant taxa. *Met* metronidazole

Interestingly, one of the most striking results following metronidazole treatment was an increase in the mean relative abundance of *L. iners* in both vaginal fluid and urine to levels above those found in healthy urine. By contrast, *L. crispatus* which is the most abundant *Lactobacillus* species in the vaginal fluid of healthy women and also present in the healthy FUM, where it constitutes UT 7 (Fig. 5), was almost absent in urine after metronidazole treatment; although, it was present at high mean relative abundance in vaginal fluid of the same patients.

OTUs that were abundant in urine, but not in vaginal fluid, were *Chitiniphagaceae*, *E. faecalis*, *E. coli*, *S. sonnei*, *S. agalactiae*, *Citrobacter murliniae* and *Propionibacterium propionicus*. With the exception of *Chitinophagaceae*, they were found both in BV and in health. Metronidazole treatment had little influence on their mean relative abundance. Thus, with the notable exception of BV-associated bacteria, most OTUs showed a complementary mean relative abundance pattern, i.e. they were either abundant in urine or in vaginal fluid samples. Metronidazole treatment did not affect those OTUs that were abundant only in urine.

## Discussion

The aim of our study was to characterize the urinary microbiota of healthy men and women and compare it to the urinary microbiota during acute BV and after antibiotic treatment in order to better understand the role of bacteria in urine for BV and its etiology. Therefore, we additionally compared microbiota in urine samples to those in vaginal fluid samples of the same women before and after metronidazole treatment of BV.

We identified nine urotypes. Interestingly, all but two were shared between men and women and were present in both BV and health. Two urotypes were found in women only. UT 7, dominated in relative abundance by *L. crispatus*, was present in healthy women, and UT 9, dominated in relative abundance by an OTU belonging to *Chitinophagaceae*, was only found in women after metronidazole treatment. Unexpectedly, no urotype characteristic of the male urinary microbiota was found. A very strong correlation between the microbiota in the vagina and male urethra and penile skin has been demonstrated for sexual partners if the woman suffered from BV [20]. In our study, male and female participants were not related. Yet, the male urine microbiota were nested inside the female microbiota and dispersed among seven of the nine urotypes found here.

The term urotype was first introduced in 2014 in a study by Pearce et al. in which they identified 6 UTs in 48 women with and without UI based on hierarchical clustering of the Euclidean distance [5]. In our study, we confirmed the UTs dominated in relative abundance by *Lactobacillus* (UT 2 and UT 7), *Gardnerella* (UT 1c), *Sneathia* (UT 1b), *Enterobacteriaceae* (UT 3) and a diverse UT (UT 8). However, our data had a higher taxonomic resolution and we were therefore able to identify the responsible species and find additional UTs, such as *L. iners* and *L. crispatus* (UT 2 and UT 7) as subtypes of the previously described *Lactobacillus* UT, *S. amnii* as responsible species for the *Sneathia* UT and *Shigella sonnei* and *E. coli* responsible for the *Enterobacteriaceae* UT. We could not identify an UT characterized by Staphylococcus, but found additional UTs that had not been detected previously. They were characterized by *P. amnii* (UT 1a), *A. vaginae* (UT 1d), *En. faecalis* (UT 4), *St. agalactiae* (UT5) and *C. murliniae* (UT6), respectively.

Our data showed that the female urinary microbiota differed in health and BV, but these changes were complex. All UTs were present in health as well as in BV. Shannon diversity decreased in BV, in contrast to the vaginal microbiota where the diversity is strongly increased in BV [23, 30–33]. Alpha-diversity was unchanged in urine of healthy women and those with BV, indicating that the shift in Shannon diversity was caused by a shift of abundance.

Oral metronidazole treatment affected the urinary microbiota further, indicated by an additional significant decrease in Shannon diversity which persisted for up to 28 days. This was found although the different UTs varied strongly in diversity and a high inter-individual variability was present, as found before [2]. The changes of the urinary microbiota of women with BV after metronidazole treatment were not unidirectional, i.e. they did not result in restoration of a healthy UT. The largest differences before and after metronidazole treatment on a cumulative level were observed for *Lactobacillus* species. *L. iners* increased after metronidazole treatment to a level that was 2.5 fold higher than during BV. In contrast, *L. crispatus* was not present during acute BV or directly after metronidazole treatment, but was the second most abundant OTU in the healthy FUM after *L. iners*. These findings are in accordance with those on the vaginal microbiota, where *L. crispatus* and *L. iners* are negatively correlated [30]. Interestingly, the importance of *Lactobacillus sp.* in the urinary microbiota during health and disease was found to be either protective or harmful depending on the disease [8]. This inconsistency may have been caused by the lack of species-level resolution. In the vaginal microbiota, *L. iners* has a controversial role whereas the beneficial role of *L. crispatus* is widely acknowledged and it is suggested as a probiotic for the prophylaxis of BV [12, 34, 35].

Oral metronidazole treatment affected the UTs present in BV in different ways. In UT 1, the relative abundance of the BV-associated bacteria *P. amnii*, *S. amnii, G. vaginalis* and *A. vagina* which dominate this UT was not affected by the antibiotic. These genera have all been detected in the FUM of women who did not suffer from an infection; the urine samples had been obtained using a transurethral catheter, i.e. these bacteria did not originate from the vagina [5, 11]. These finding suggest that the urethra and bladder might act as a reservoir for BV-associated bacteria. At the same time, in UT 1 patients, a number of BV-related bacteria were significantly enriched after antibiotic treatment, including BVAB2, BVAB3 and Megasphaera. These taxa have not been detected in the above mentioned studies and thus may have originated from the vagina, where they are commonly found during BV [33].

In UT 2 patients, *G. vaginalis* was effectively eliminated by metronidazole treatment, while *L. iners* was enriched. This species was one of the first to be detected in transurethral catheter urine samples [6]. In UT 3, *G. vaginalis* was also eliminated by metronidazole treatment, and here *Shigella dysenteriae* was enriched. Even though taxonomic classification based on the 16S rRNA sequence within the Enterobacteriaceae family is challenging, this OTU assigned to *Sh. dysenteriae* belongs to the family of Enterobacteriaeceae, and therefore, we state that metronidazole treatment did not result in a UT shift.

*Lactobacillus* species are a major part of the FUM [2] as well as of the vaginal microbiota [36, 37], and it remains to be clarified if they reside in the urethra and bladder and contribute to recurrence. Some of the OTUs found here are also known from other body sites, e.g. *E. coli* and *Proteus mirabilis* from the rectum or *Staphylococcus aureus* from the skin and might have entered the samples through smear infection. Even though they were present, they did not play a major role in this analysis due to their low abundance. The increase in mean relative abundance of *L. iners* was the most remarkable change of the urine microbiota after oral metronidazole treatment and it similarly occurred in vaginal fluid [23] suggesting that this species might reside in the vagina. However, *L. iners* was detected in transurethral catheter urine samples [6]. Detailed analyses of samples obtained directly from the bladder and urethra of healthy men and women are required to clarify whether *L. crispatus*, *L. iners* or both are residents of the urinary tract and might contribute to recolonizing the vagina after episodes of BV. Currently, it is hypothesized that pathogens residing in the vagina can colonize the urethra and thus cause urinary tract infections [38]; given the presence of BV-related bacteria in the urethra and bladder of healthy women and in the urethra of healthy men, the converse route could also be possible and should be investigated in the future. Moreover, certain urinary tract microbiota might have a protective role [8] thereby preventing bacterial vaginosis or its recurrence.

Oral application of metronidazole for treatment of BV results in systemic absorption, while localized vaginal application at high doses shows the same treatment success but only little systemic absorption [39]. Therefore, the impact of localized metronidazole application on the urinary microbiota should be minimal. Analysing the effect of oral vs. vaginal metronidazole treatment on the urinary microbiota might give further insights into the role of the urinary microbiota in BV etiology and if modifications of the urinary microbiota by systemic metronidazole treatment is beneficial or detrimental for BV recurrence.

## Conclusions

This study indicates that the male urine microbiota formed a subgroup of the female urinary microbiota rather than a separate cluster, suggesting that it was not strongly influenced by the distal regions of the urogenital tract. Oral application of metronidazole in women with BV caused massive changes in urine microbiota composition, but it did not restore a healthy community. While some pathogens were eradicated, others even increased in relative abundance, suggesting strongly different susceptibility to the antibiotic. The data suggest that the urinary tract harbors distinct microbiota that may contribute to BV recurrence but might also serve a protective role.

## Additional files

Additional file 1: Figure S1. Principle coordinate analysis of all samples from healthy participants. Samples are colored according to antibiotic intake in the 10 days before urine sampling. (TIFF 143 kb)

Additional file 2: Table S1. List of raw and final sequencing results as well as all metadata. (XLSX 2040 kb)

Additional file 3: Figure S2. Rarefaction curves of all samples. Samples were grouped according to healthy male, healthy female, inclusion/exclusion during acute BV and the time point after metronidazole treatment. The *x* axis was cut at the mean value of sequencing depth. (TIFF 969 kb)

Additional file 4: Figure S3. Determination of resampling efficiency. Resampling to the lowest sequencing depth of 2196 reads was performed 20 times for 4 randomly chosen samples with low to high sequencing depth: (A) 5042 reads, (B) 17,477 reads, (C) 43,494 reads and (D) 69,765 reads. The standard error (standard deviation of the mean) is indicated. (TIFF 322 kb)

Additional file 5: Figure S4. Urinary microbial communities before and after metronidazole treatment. (A) Microbial profiles for every women. (B) Mean relative abundance of the study set. The 19 most abundant OTUs are shown and all others (<1.2% rel. abundance each) are summarized as “others”. (TIFF 2422 kb)

Additional file 6: Figure S5. Individual progression of the urinary and vaginal fluid microbiota before and after treatment with metronidazole. The 20 most abundant OTUs in urine and vaginal fluid are shown and all others are summarized as “others”. (TIFF 2701 kb)
