# Supplementary figures and images for: The urinary microbiota of men and women and its changes in women during bacterial vaginosis and antibiotic treatment
Source: Microbiome. 2017 Aug 14;5:99. doi: 10.1186/s40168-017-0305-3 (PMC5554977; doi:10.1186/s40168-017-0305-3)

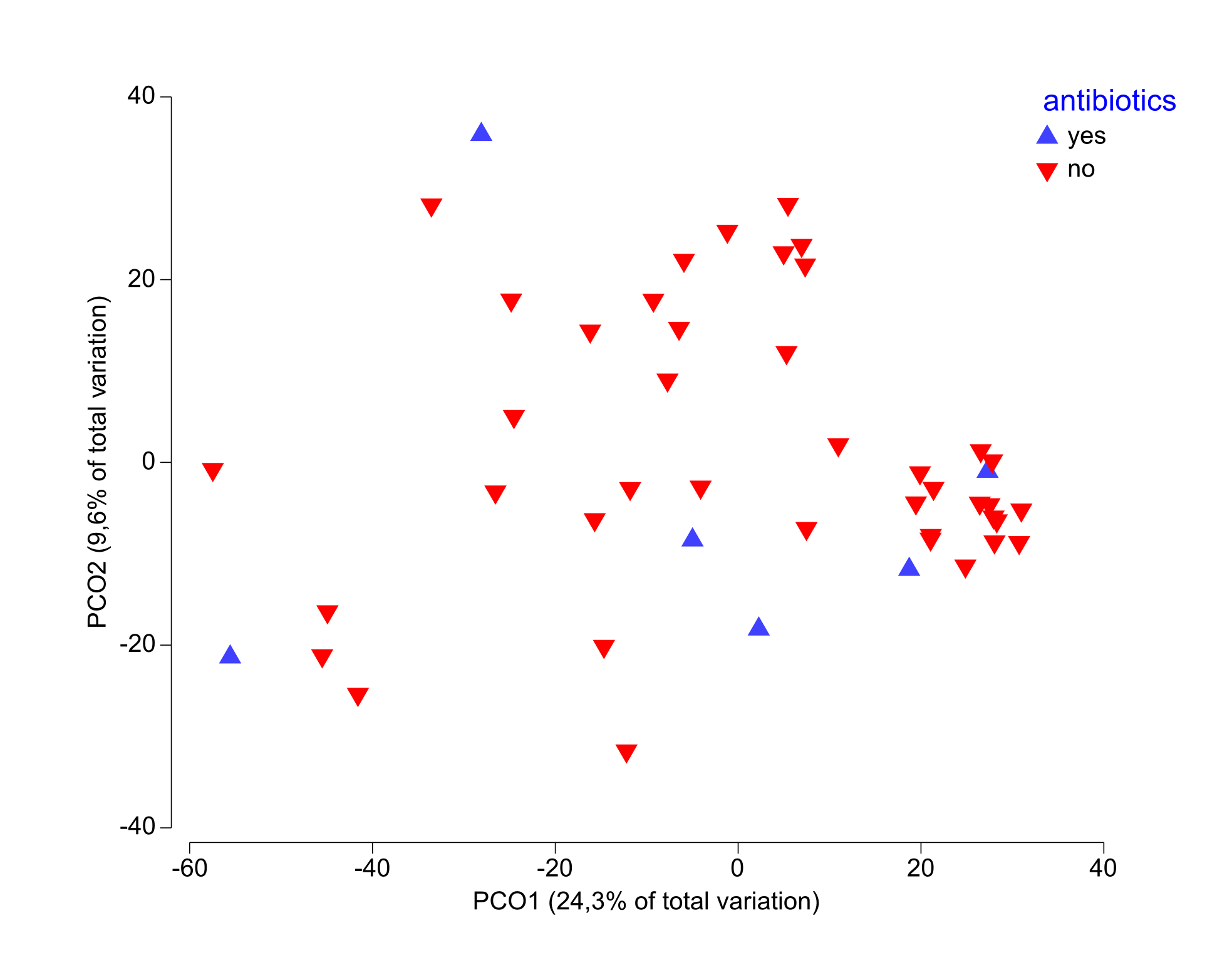

Supplement: Supplementary file 1 — Principle coordinate analysis of all samples from healthy participants. Samples are colored according to antibiotic intake in the 10 days before urine sampling. (TIFF 143 kb) [file 40168_2017_305_MOESM1_ESM.tif]

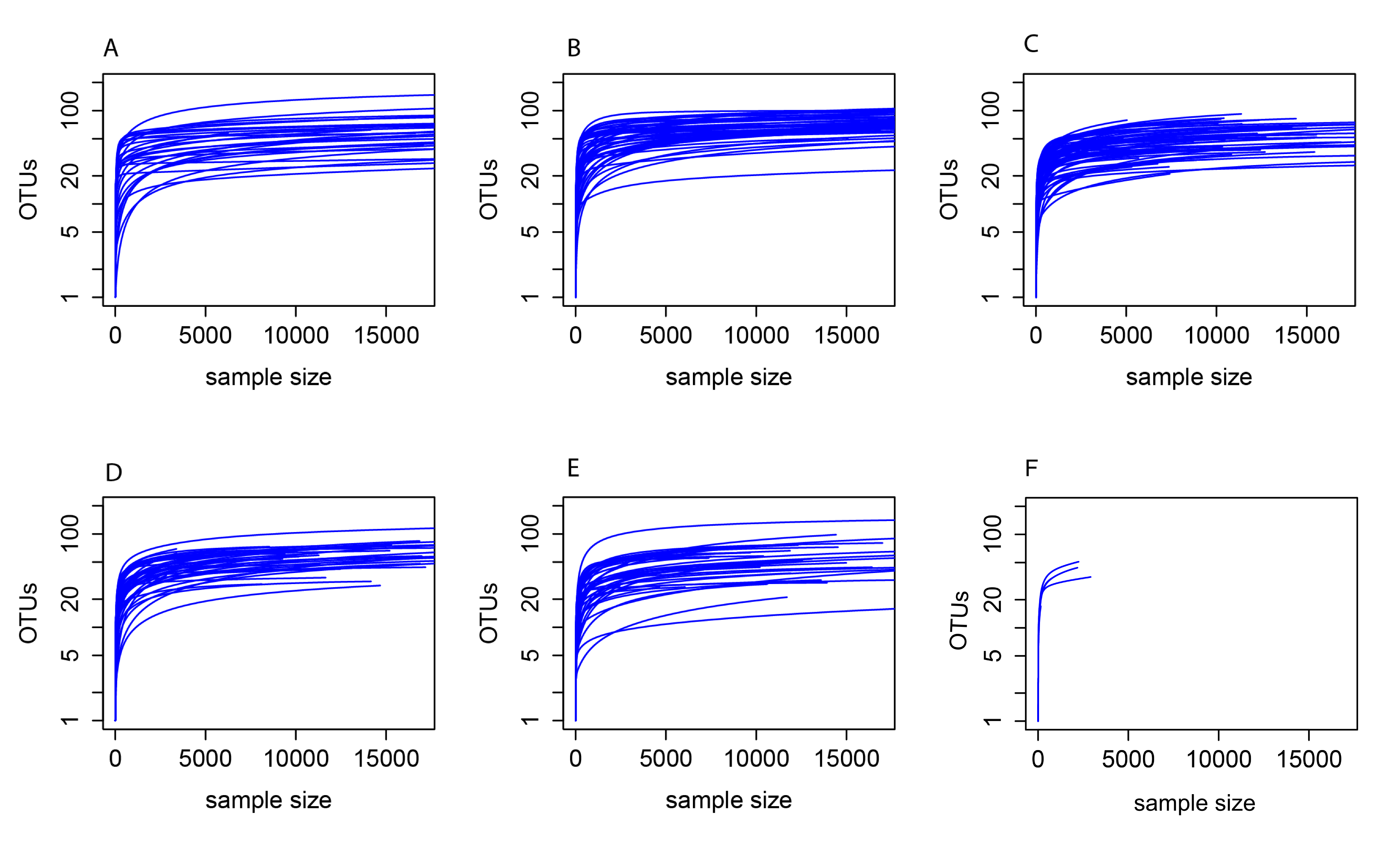

Supplement: Supplementary file 3 — Rarefaction curves of all samples. Samples were grouped according to healthy male, healthy female, inclusion/exclusion during acute BV and the time point after metronidazole treatment. The x axis was cut at the mean value of sequencing depth. (TIFF 969 kb) [file 40168_2017_305_MOESM3_ESM.tif]

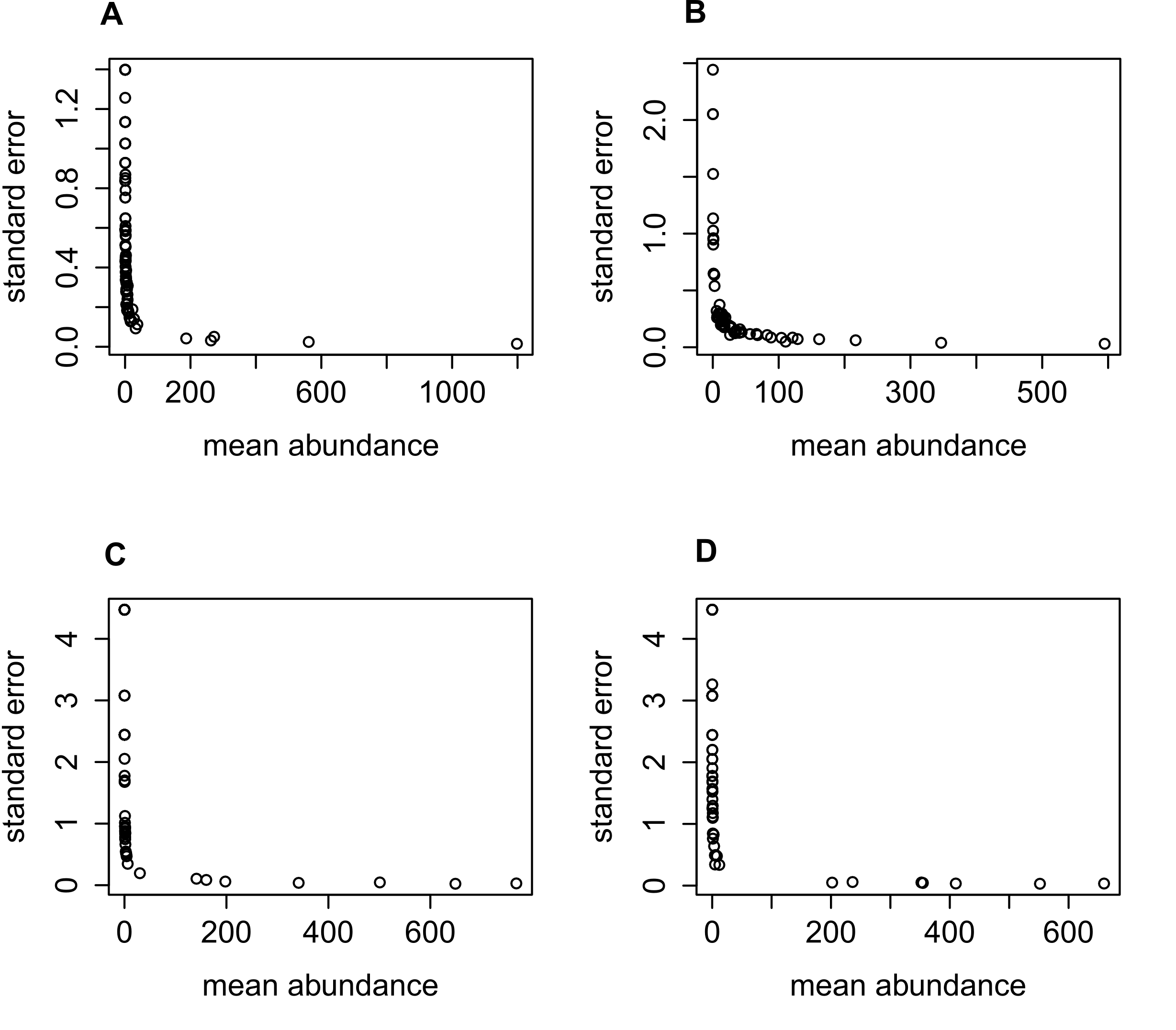

Supplement: Supplementary file 4 — Determination of resampling efficiency. Resampling to the lowest sequencing depth of 2196 reads was performed 20 times for 4 randomly chosen samples with low to high sequencing depth: (A) 5042 reads, (B) 17,477 reads, (C) 43,494 reads and (D) 69,765 reads. The standard error (standard deviation of the mean) is indicated. (TIFF 322 kb) [file 40168_2017_305_MOESM4_ESM.tif]

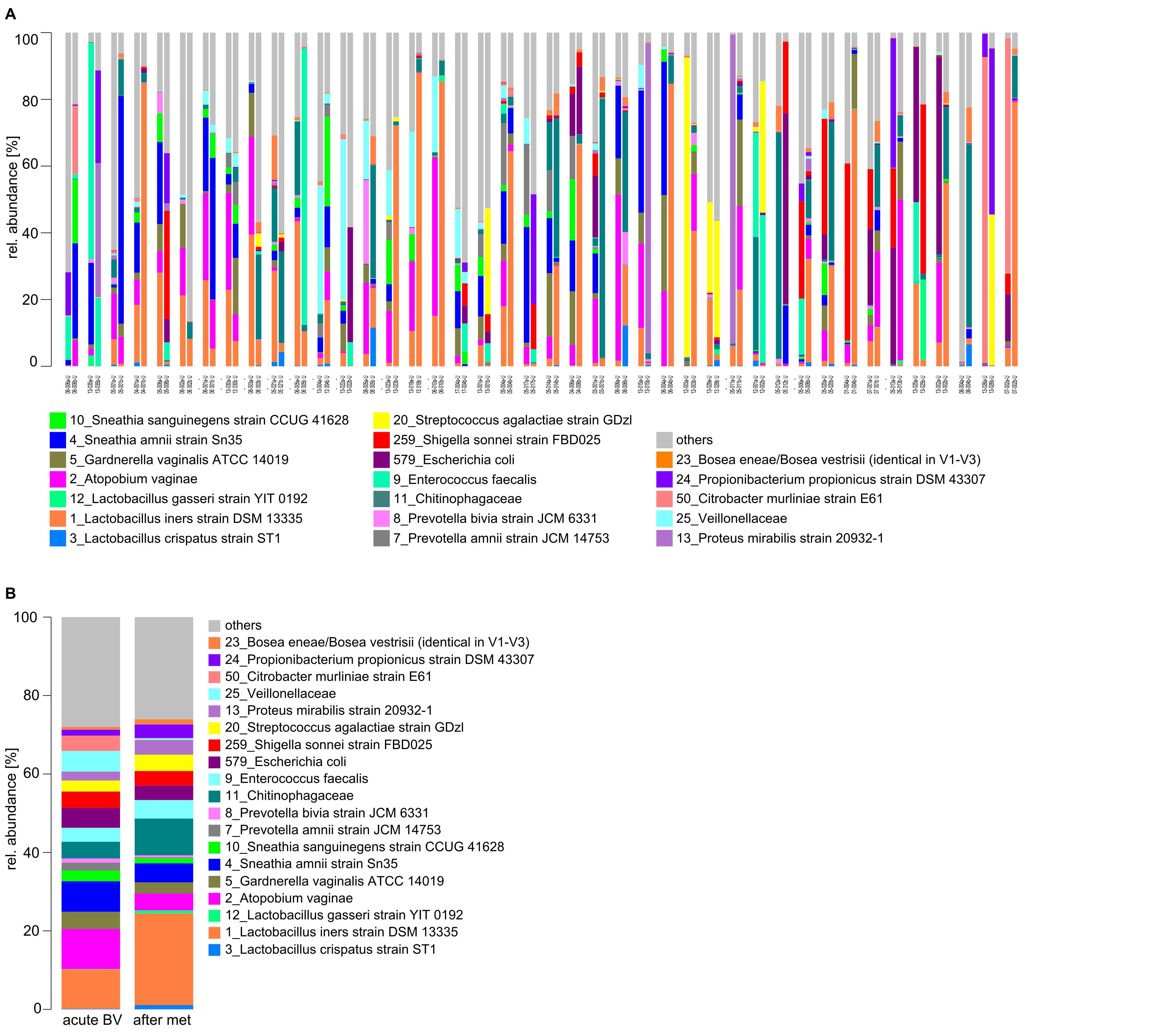

Supplement: Supplementary file 5 — Urinary microbial communities before and after metronidazole treatment. (A) Microbial profiles for every women. (B) Mean relative abundance of the study set. The 19 most abundant OTUs are shown and all others (<1.2% rel. abundance each) are summarized as “others”. (TIFF 2422 kb) [file 40168_2017_305_MOESM5_ESM.tif]

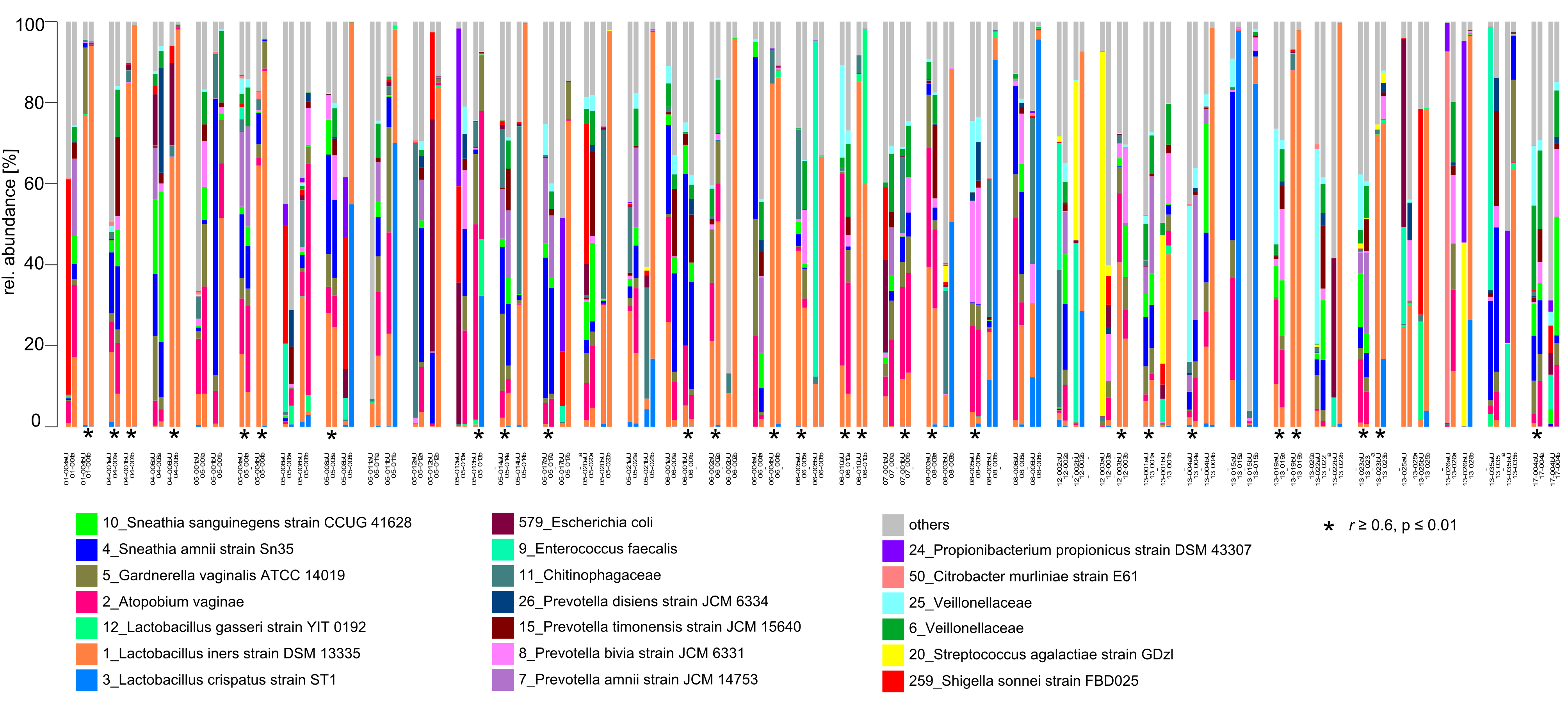

Supplement: Supplementary file 6 — Individual progression of the urinary and vaginal fluid microbiota before and after treatment with metronidazole. The 20 most abundant OTUs in urine and vaginal fluid are shown and all others are summarized as “others”. (TIFF 2701 kb) [file 40168_2017_305_MOESM6_ESM.tif]
